# Long-term combined application of manure and chemical fertilizer sustained higher nutrient status and rhizospheric bacterial diversity in reddish paddy soil of Central South China

**DOI:** 10.1038/s41598-018-34685-0

**Published:** 2018-11-08

**Authors:** Xinwei Cui, Yangzhu Zhang, Jusheng Gao, Fuyuan Peng, Peng Gao

**Affiliations:** 1 College of Resources and Environment, Hunan Agricultural University, Changsha, 410128 China; 2 Red Soil Experimental Station of Chinese Academy of Agricultural Sciences, Qiyang, Hunan 426182 China; 3 Institute of Agro-Environment and Ecology, Hunan Academy of Agricultural Sciences, Changsha, 410125 China

## Abstract

Bacteria, as the key component of soil ecosystems, participate in nutrient cycling and organic matter decomposition. However, how fertilization regime affects the rhizospheric bacterial community of reddish paddy soil remains unclear. Here, a long-term fertilization experiment initiated in 1982 was employed to explore the impacts of different fertilization regimes on physicochemical properties and bacterial communities of reddish paddy rhizospheric soil in Central South China by sequencing the 16S rRNA gene. The results showed that long-term fertilization improved the soil nutrient status and shaped the distinct rhizospheric bacterial communities. Particularly, chemical NPK fertilizers application significantly declined the richness of the bacterial community by 7.32%, whereas the application of manure alone or combined with chemical NPK fertilizers significantly increased the biodiversity of the bacterial community by 1.45%, 1.87% compared with no fertilization, respectively. Moreover, LEfSe indicated that application of chemical NPK fertilizers significantly enhanced the abundances of *Verrucomicrobia* and *Nitrospiraceae*, while manure significantly increased the abundances of *Deltaproteobacteria* and *Myxococcales*, but the most abundant *Actinobacteria* and *Planctomycetes* were detected in the treatment that combined application of manure and chemical NPK fertilizers. Furthermore, canonical correspondence analysis (CCA) and the Mantel test clarified that exchangeable Mg^2+^ (E-Mg^2+^), soil organic carbon (SOC) and alkali-hydrolyzable nitrogen (AN) are the key driving factors for shaping bacterial communities in the rhizosphere. Our results suggested that long-term balanced using of manure and chemical fertilizers not only increased organic material pools and nutrient availability but also enhanced the biodiversity of the rhizospheric bacterial community and the abundance of *Actinobacteria*, which contribute to the sustainable development of agro-ecosystems.

## Introduction

Fertilization has been extensively used as a common management practice to maintain soil fertility and crop productivity^1^. Chemical fertilizers are extremely attractive and commonly used for their high nutrient concentration, easy availability and convenient transportation and application^2^. In the pursuit of economic growth and food production, increasing amounts of chemical fertilizers have been applied in agroecosystems all over the world^3,4^, which has resulted in serious degradation of soil physicochemical properties and productivity deterioration^5,6^. Alternatively, organic fertilizers are derived from animal and/or plant matter, which can modify soil physicochemical conditions because of abundant organic matter and balanced nutrients^7–9^. Unfortunately, the lower nutrient content and nutrient release rate of organic fertilizers make them less likely to meet crop requirements, what’s more, compared with chemical fertilizers, the higher amount and inconvenient usage of organic fertilizers resulted in a less prevalence. Nonetheless, combined using of organic and chemical fertilizers has been proven as an effective approach to improve soil fertility, crop yields and environmental quality compared with the single use of either of them^10–12^.

Microorganisms are the key components of the soil ecosystems and involve in nutrient cycling, energy flow, and organic matter degradation^13^. Bacteria, as the most abundant and diverse group of soil microorganisms^14^, which participate in biogeochemical nutrient cycling and organic matter decomposition processes^2^ and maintain ecological balance^15^. Clearly understanding the responsive shifts of bacterial community structure and composition to different fertilization regimes is vital for developing better fertilization practices and for further improving soil fertility and function^2^. High-throughput sequencing technologies had provided important ways to determine microbial information in recent years, and many studies had generated more valuable soil microbial information through experiments involving long-term fertilization practices. However, most had focused on dryland soil agro-ecosystems^16–19^, just a few studies had reported microbial community characteristics in paddy soils. For example, the study by Daquiado *et al*.^20^ was performed in silty mixed mesic Typic Haplaquepts and specific climates, while the observation of Chen *et al*.^2^ focused on the effects of different ratios of organic and inorganic fertilizers, both of them viewed on bulk soil bacteria. Actually, the microbial community in the rhizosphere had more important impacts on the growth and health of plants than that in bulk soil^21,22^. Therefore, exploring the rhizospheric microbial community is vital for understanding the interactions among soil, microbes and the host plants under long-term fertilization, which can help identify better fertilizer regimes and plant breeding methods for heavily fertilized cropland^23^.

In this study, a long-term field experiment with four different fertilization regimes in a rice-rice cropping rotation system, established in the Red Soil Experimental Station of the Chinese Academy of Agricultural Sciences since 1982, was conducted to investigate the influences of fertilization regime on the rhizospheric bacterial community structure and composition in the Quaternary reddish paddy soil of Central South China. The objectives were (i) to identify the effects of different fertilization regimes on the rhizospheric bacterial diversity and richness of reddish paddy soil; (ii) to explore the relative abundances of dominant bacterial phyla under different fertilization regimes; (iii) to screen specific biomarkers of different fertilization regimes under different bacterial taxonomic levels; and (iv) to clarify the key driving factors for shaping bacterial communities in the reddish paddy soil of Central South China.

## Results

### Effects of different fertilization regimes on soil properties

Long-term fertilization dramatically altered soil physicochemical properties (Table 1). The soil pH value significantly (*p* < 0.05) decreased by 3.55% under NPK treatment but significantly (*p* < 0.05) increased by 2.88%, 2.54% in response to manure treatments (M and NPKM) compared with NF treatment, respectively. Furthermore, long-term fertilization showed significantly (*p* < 0.05) increased SOC(16.42∼79.93%), AN(29.91∼87.13%), AP(83.53 ∼335.43%), AK(9.04∼80.88%), E-Ca^2+^ (12.96∼19.93%) and E-Mg^2+^ (5.62∼15.73%) compared with no fertilization, and in particular, significantly (*p* < 0.05) higher levels of SOC, AN, AP, and AK were observed in NPKM treatment than in NPK or M treatment; The opposite trend was observed for E-Mg^2+^. SOC/TN slightly increased by 0.23%, 9.31% in NPK and M treatments, but it significantly (*p* < 0.05) increased by 23.95% in NPKM treatment compared with no fertilization. In addition, SOC/TN in NPKM treatment was significantly (*p* < 0.05) increased by19.14%, 11.81% than in NPK or M treatment.

**Table 1:** The properties of paddy soil subjected to long-term different fertilization regimes.

Treatment	pH	SOC (g/kg)	AN(mg/kg)	AP(mg/kg)	AK(mg/kg)	E-Ca^2+^(cmol/kg)	E-Mg^2+^(cmol/kg)	SOC/TN
NF	5.91 ± 0.01 b	13.70 ± 0.72 d	113.83 ± 1.24 d	10.02 ± 0.39 c	98.74 ± 4.86 c	3.01 ± 0.08 c	0.89 ± 0.00 c	8.81 ± 0.39 b
NPK	5.70 ± 0.03 c	15.95 ± 0.89 c	147.88 ± 11.41 c	21.21 ± 1.86 b	107.67 ± 2.83 c	3.40 ± 0.09 b	1.02 ± 0.01 a	8.83 ± 0.24 b
M	6.08 ± 0.02 a	20.45 ± 0.56 b	182.18 ± 10.41 b	18.39 ± 1.38 b	164.64 ± 7.95 b	3.48 ± 0.07 ab	1.03 ± 0.00 a	9.63 ± 0.56 b
NPKM	6.06 ± 0.01 a	24.65 ± 0.72 a	213.01 ± 5.85 a	43.63 ± 2.52 a	178.60 ± 7.37 a	3.61 ± 0.09 a	0.94 ± 0.00 b	10.92 ± 0.72 a

Date are presented as the Mean ± Standard Deviation (n = 3). Different letters within columns followed by indicate significance at *P* < 0.05 according to Duncan’s test. NF, no fertilization treatment; NPK, chemical NPK fertilizers treatment; M, composted manure treatment; NPKM, chemical NPK fertilizers plus composted manure treatment. SOC, soil organic carbon; AN, alkali-hydrolyzable nitrogen; AP, available phosphorus; AK, available potassium; E-Ca^2+^, exchangeable Ca^2+^; E-Mg^2+^, exchangeable Mg^2+^; SOC/TN, the ratio of soil organic carbon to soil total nitrogen.

### General analyses of the sequencing data

Across all soil samples, a total of 985,898 sequence reads were successfully elicited. After removing short and low-quality reads, singletons, replicates and chimeras, 601,336 sequences, ranging from 41,702 to 57,123 per sample, were retained. Based on 97% similarity, a total of 5585 OTUs, ranging from 2279 to 2616 per sample, were obtained across all samples (Table S1). Among the total sequences, ~99.4% were classified as bacteria, with 62 phyla, 146 classes, 185 orders and 303 families.

A rarefaction analysis showed that the number of OTUs observed for 16S did not reach saturation (Fig. S1), which indicated that the sequencing capability was not large enough to capture the complete biodiversity of these communities, as the curves did not reach a plateau by increasing sample size, a result similar to several previous observations^16,20^. However, the data were sufficient to show differences among the treatments and to reveal the influences of different long-term fertilization regimes on the soil bacterial community.

### Richness and biodiversity of the bacterial community in the rhizosphere

The coverage indices of all treatments were more than 0.98, which also indicated that the sequencing capability was large enough to capture most of the bacterial community characteristics of each treatment (Table 2). The number of observed OTUs in NPK treatment was significantly (*p* < 0.05) decreased by 5.78%, 7.00%, 8.82% than in NF, M and NPKM treatments, but less significant differences were detected among the latter three treatments (2434.33∼2515.67). Meanwhile, the NPKM treatment had the highest Shannon diversity index (9.83), followed by M treatment (9.79), there was no significant difference between NPKM and M treatments, but both of them were significantly (*p* < 0.05) enhanced by 2.61∼2.19%, 1.87∼1.45% than NPK (9.58) and NF (9.65) treatments, respectively. In addition, the richness index Chao1 in NPK treatment was significantly (*p* < 0.05) decreased by 7.32%, 7.41%, 8.67% than in NF, M and NPKM treatments, and slight differences were observed among NF, M and NPKM treatments (2512.10∼2549.42).

**Table 2:** Estimated number of observed OTUs, biodiversity, richness and coverage across treatments.

Treatment	Observed OTUs	Shannon	Chao1	Coverage
NF	2434.33 ± 40.38 a	9.65 ± 0.05 b	2512.10 ± 69.80 a	0.9874 ± 0.0025 a
NPK	2293.67 ± 16.80 b	9.58 ± 0.09 b	2328.27 ± 24.73 b	0.9906 ± 0.0025 a
M	2466.33 ± 28.57 a	9.79 ± 0.01 a	2514.69 ± 53.88 a	0.9890 ± 0.0029 a
NPKM	2515.67 ± 89.05 a	9.83 ± 0.08 a	2549.42 ± 107.85 a	0.9903 ± 0.0045 a

Date are presented as the Mean ± Standard Deviation (n = 3). Different letters within columns followed by indicate significance at *P* < 0.05 according to Duncan’s test. NF, no fertilization treatment; NPK, chemical NPK fertilizers treatment; M, composted manure treatment; NPKM, chemical NPK fertilizers plus composted manure treatment. Observed OTUs, observed operational taxonomic units; Shannon, nonparametric Shannon diversity index; Chao1, richness of the Chao1 estimator; Coverage, Good’s nonparametric coverage estimator.

### Effects of different fertilization regimes on bacterial community structures in the rhizosphere

The unweighted pair group method using arithmetic averages (UPGMA) was used to compare the phylogenetic relationships of bacterial communities based on the weighted UniFrac similarity index of OTU abundance, and the result is presented in Fig. S2. In general, the 12 investigated communities (three replicates for each of the 4 treatments) were divided into two major groups: For NF and NPKM treatments, the three replicate samples of each treatment were closest and grouped together, the NF and NPKM were further grouped together; for M and NPK treatments, a total of the 6 samples were roughly grouped together, and the two treatments were not separated clearly.

**Figure 1:**
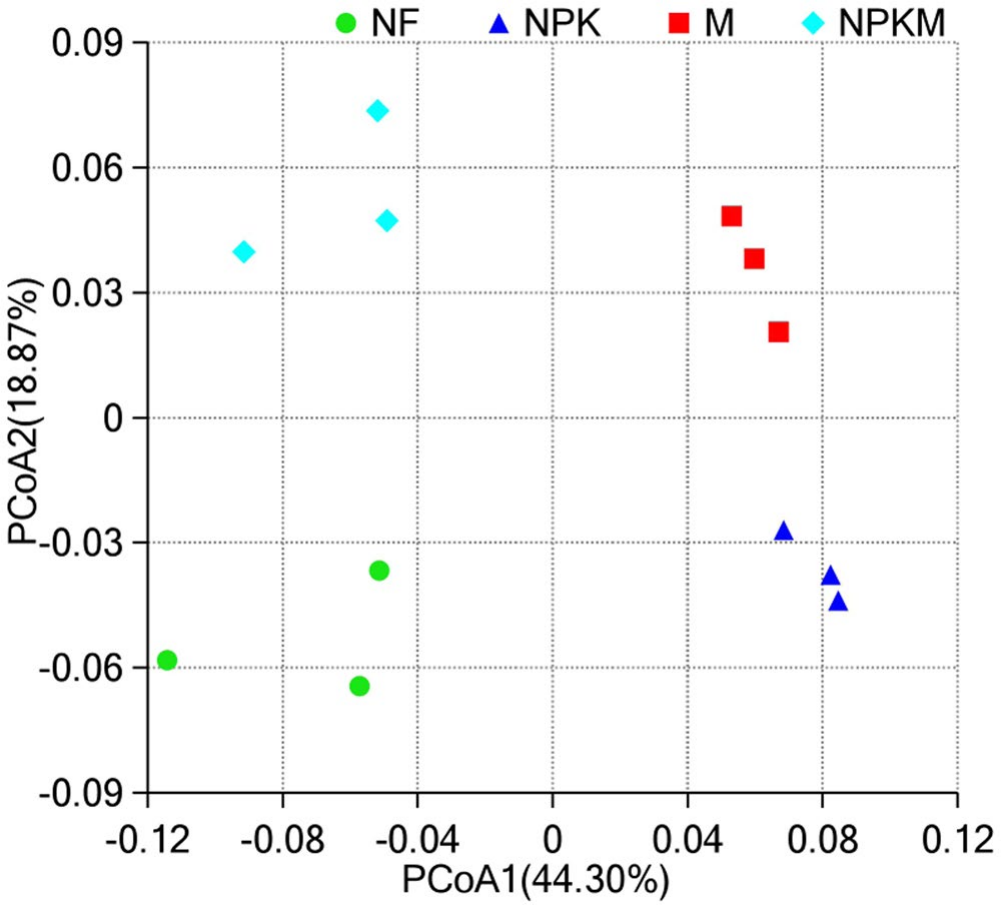
Principal coordinate analysis (PCoA) based on the distance matrix calculated using the weighted UniFrac algorithm of bacterial communities in 12 soil samples subjected to long-term different fertilization regimes. Note: NF, no fertilization treatment; NPK, chemical NPK fertilizers treatment; M, composted manure treatment; NPKM, chemical NPK fertilizers plus composted manure treatment.

Furthermore, two-dimensional principal coordinate analysis (PCoA) based on the weighted UniFrac distance clearly revealed that the soil bacterial communities varied among the different fertilization treatments (Fig. 1). The four different fertilization treatments are clearly separated and distributed in four quadrants along the first and second components (PCoA1, PCoA2), and the first two axes can explain ~63.17% of the observed variance in total, which indicates that the soil bacterial community structures of the four treatments may maintain great differences, though more information is needed.

### Effects of different fertilization regimes on bacterial community compositions in the rhizosphere

**Figure 2:**
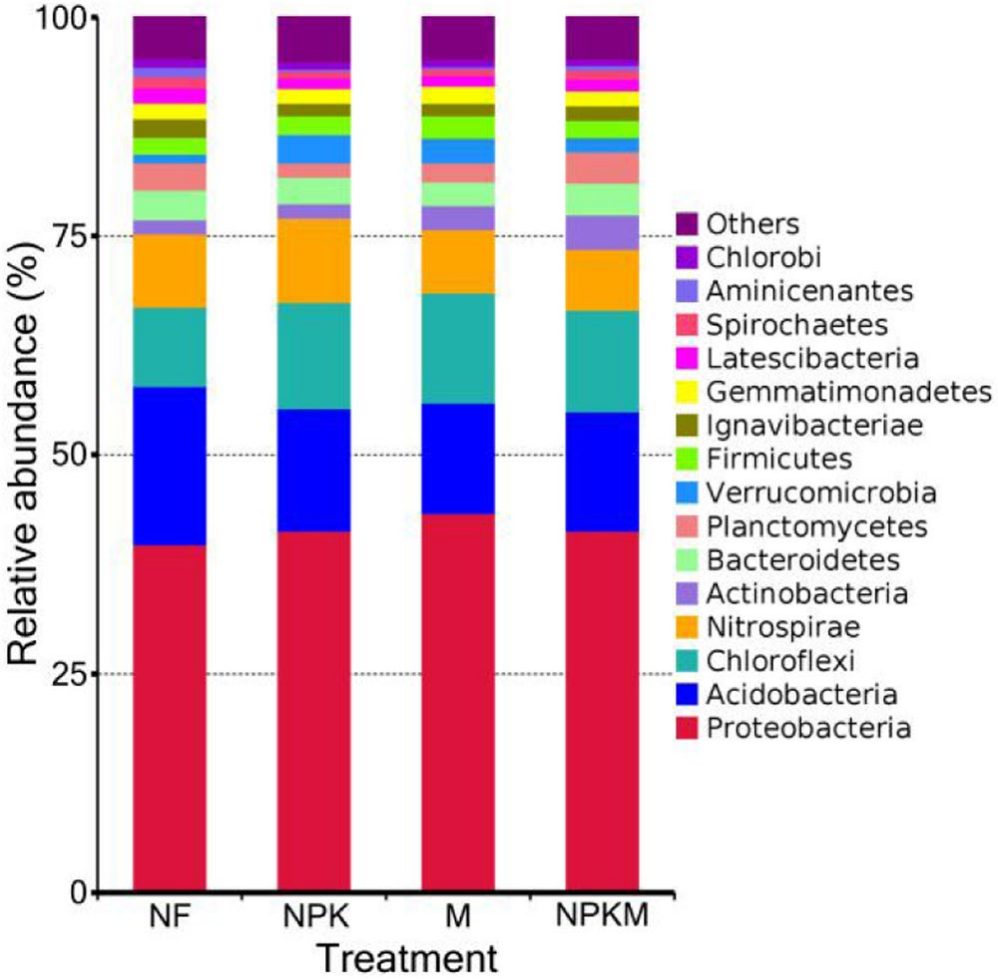
The relative abundances (%) of the top 15 bacterial phyla present in the 4 treatments. Note: NF, no fertilization treatment; NPK, chemical NPK fertilizers treatment; M, composted manure treatment; NPKM, chemical NPK fertilizers plus composted manure treatment.

The top 4 dominant phyla across all samples were *Proteobacteria*, *Acidobacteria*, *Chloroflexi* and *Nitrospirae*, which accounted for more than 70% of the relative abundance of the bacterial communities (Fig. 2, Fig. S3). Among these dominant phyla, *Proteobacteria* and *Chloroflexi* were most abundant in M treatment (*Proteobacteria* 43.39%, *Chloroflexi* 12.62%) but least abundant in NF treatment (*Proteobacteria* 39.83%, *Chloroflexi* 9.07%). Conversely, *Acidobacteria*, as the second-most abundant phylum, was most abundant in NF treatment (18.13%) but least abundant in M treatment (12.52%). Differently, *Nitrospirae* sustained most abundant in NPK treatment (9.65%) and least abundant in NPKM treatment (6.96%).

**Figure 3:**
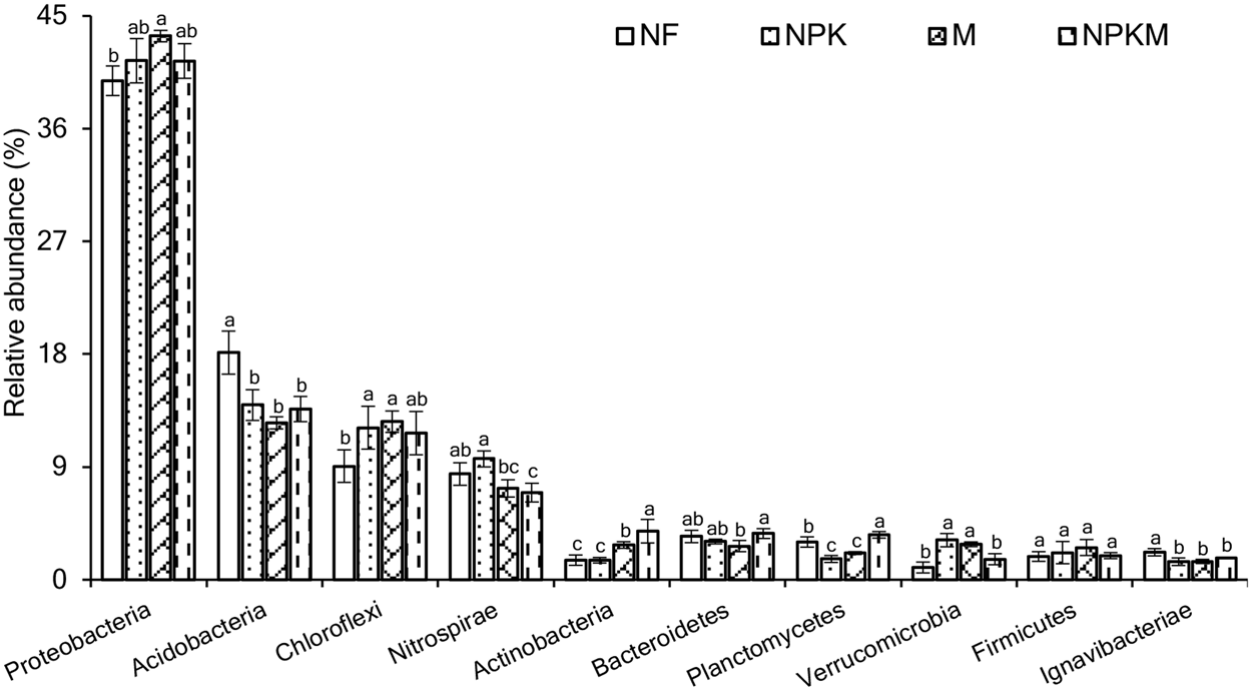
Comparison of the relative abundances of the top 10 dominant bacteria phyla across treatments. Bars indicate standard error (n = 3). Different letters above columns within the same species indicate significance at *P* < 0.05 according to Duncan’s test. NF, no fertilization treatment; NPK, chemical NPK fertilizers treatment; M, composted manure treatment; NPKM, chemical NPK fertilizers plus composted manure treatment.

Furthermore, an analysis of variance was employed to explore the differences of relative abundances in the top 10 bacterial phyla (Fig. 3). For NF treatment, the abundances of *Proteobacteria*, *Chloroflexi*, and *Verrucomicrobia* were decreased by 3.82∼8.22%, 22.52∼28.17%, 40.35∼69.03% than in other treatments, whereas the abundances of *Acidobacteria* and *Ignavibacteriae* were significantly (*p* < 0.05) increased by 30.00∼43.73%, 27.25∼52.66% than in other treatments, respectively. For NPK treatment, the abundances of *Actinobacteria*, *Planctomycetes* and *Ignavibacteriae* were declined by 0.58∼59.74%, 21.75∼53.55%, 1.62∼34.49% as compared with other treatments, whereas the abundances of *Nitrospirae* and *Verrucomicrobia* increased by 14.34∼38.70%, 11.44∼222.91% than in other treatments. For M treatment, the abundances of *Acidobacteria* and *Bacteroidetes* were decreased by 8.06∼30.91%, 12.06∼27.27% than in other treatments, and the abundances of *Proteobacteria*, *Chloroflexi* and *Firmicutes* were increased by 4.73∼8.95%, 3.96∼39.21%, 18.72∼38.51% than in other treatments. For NPKM treatment, the abundance of *Nitrospirae* was declined by 4.61∼27.90% than that in other treatments, and the abundances of *Actinobacteria*, *Bacteroidetes* and *Planctomycetes* were enhanced by 39.52∼148.40%, 6.71∼37.49%, 18.79∼115.28% than in other treatments, what’s more, the abundances of *Actinobacteria* and *Planctomycetes* were significantly higher than those in other treatments.

### Biomarkers of different treatments subjected to different long-term fertilization regimes

**Figure 4:**
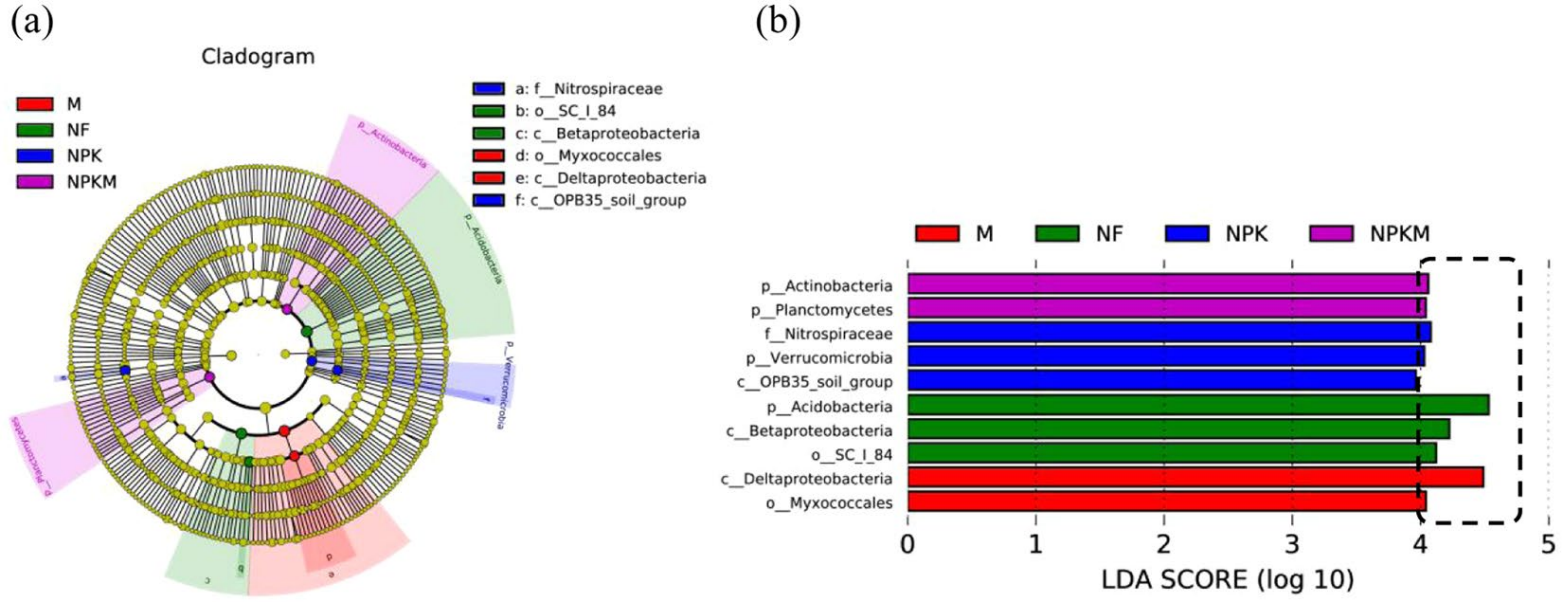
LEfSe identified the most differentially abundant taxa across treatments. (**a**) Taxonomic cladogram obtained from the LEfSe of 16S rDNA sequences (relative abundance ≥0.5%). Biomarker taxa are highlighted by coloured circles and shaded areas (NF, NPK, M and NPKM treatments are shown in green, blue, red and purple, respectively). Each circle’s diameter reflects the abundance of that taxa in the community. (**b**) The taxa whose abundance differed among the NF (green bars), NPK (blue bars), M (red bars) and NPKM (purple bars) treatments. The cutoff value of ≥4.0 used for the linear discriminant analysis (LDA) is shown. NF, no fertilization treatment; NPK, chemical NPK fertilizers treatment; M, composted manure treatment; NPKM, chemical NPK fertilizers plus composted manure treatment.

To identify the specific bacterial taxa associated with different fertilization levels, we compared the bacterial communities in NF, NPK, M and NPKM treatments using linear discriminant analysis (LDA) effect size (LEfSe). The cladogram representative of the structure of the microbial communities and their predominant bacteria is shown in Fig. 4. The greatest differences (LDA ≥ 4) in taxa among the four communities are displayed. A total of 9 biomarkers were screened under different taxonomic levels across the 4 treatments. The relative abundances of the class *Deltaproteobacteria* and order *Myxococcales* were dramatically higher in M treatment than in other 3 treatments, and they were considered as the biomarkers of M treatment. Similarly, the phylum *Acidobacteria*, class *Betaproteobacteria* and order *Sc-I-84* were considered as the biomarkers of NF treatment, the phylum *Verrucomicrobia* and family *Nitrospiraceae* were regarded as the biomarkers of NPK treatment, and the phyla *Actinobacteria* and *Planctomycetes* were deemed as the biomarkers of NPKM treatment.

### Relationships between soil properties and bacterial communities

**Figure 5:**
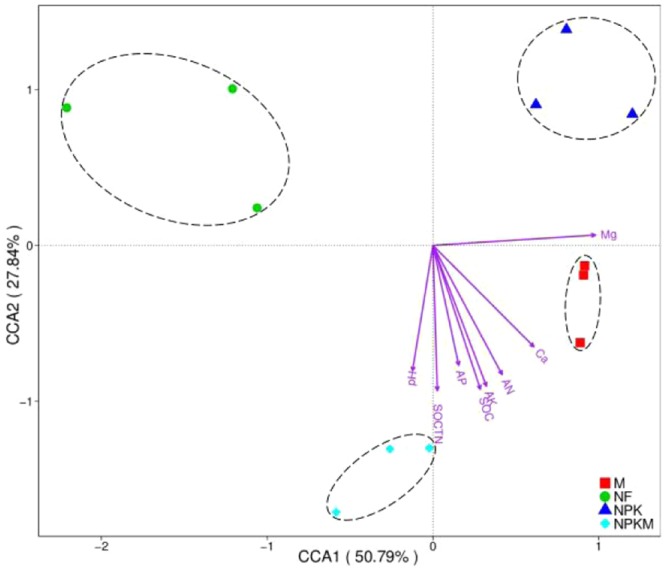
Canonical correspondence analysis (CCA) based on the relative abundance of bacterial phyla and selected soil chemical properties among different fertilization treatments. Note: NF, no fertilization treatment; NPK, chemical NPK fertilizers treatment; M, composted manure treatment; NPKM, chemical NPK fertilizers plus composted manure treatment. SOC, soil organic carbon; AN, alkali-hydrolyzable nitrogen; AP, available phosphorus; AK, available potassium; Ca, exchangeable Ca^2+^; Mg, exchangeable Mg^2+^; SOC/TN, the ratio of soil organic carbon to soil total nitrogen.

At the phylum level, canonical correspondence analysis (CCA) was employed to detect the relationships between soil properties and bacterial communities based on the relative abundances of species, which revealed that the first and second CCA components were able to explain 78.63% of the total bacterial variation (Fig. 5). The first component (CCA1), explaining 50.79% of the total variation of bacterial phyla, separated the M and NPKM treatments from NPK and NF treatments, respectively. The second component (CCA2), explaining 27.84% of the total variation of bacterial phyla, separated the NF treatment from NPK treatment. Moreover, CCA identified that different environmental variables had different impacts on the overall bacterial community, with an order as follows: E-Mg^2+^ > SOC, AK, AN, E-Ca^2+^, SOC/TN> pH, AP.

Furthermore, the Mantel test was employed to investigate Spearman’s correlated relationships between the relative abundances of bacterial phyla and the soil properties, which indicated that a series of properties, including SOC, AN, AP, and E-Mg^2+^, were significantly (*p* < 0.01) correlated with the bacterial community composition in reddish paddy soil. Moreover, the pH, AK, and E-Ca^2+^ were also significantly (*p* < 0.05) correlated with the bacterial community composition, but the SOC/TN was observed to slightly affect the bacterial community composition.

## Discussion

In this study, long-term application of composted manure either alone or combined with chemical NPK fertilizers significantly enhanced the soil pH value, whereas single application of chemical NPK fertilizers significantly declined the pH value compared with no fertilization treatment (Table 1), which was similar with previous studies^2,24^. Above those indicated that different response mechanisms of pH to fertilization regimes, guessing that manure application increased soil pH due to the liming effect of added organic matter and carbonates^25^, but single application of chemical NPK fertilizers decreased the pH for the nitrification processes that converting mineral fertilizer ammonium to nitrate^26^. Moreover, long-term fertilization (NPK, M, NPKM) increased the soil nutrient status in different degrees, among them, combined application of manure and chemical NPK fertilizers can effectively improve the concentrations of SOC, AN, AP, AK, and E-Ca^2+^ (Table 1), which in accordance with other long-term experiments in various ecosystems^18,20,27^. All these indicated that long-term balanced using of manure and chemical fertilizers increased organic material pools and nutrient availability and further improved the physical environment of soil and the yield of rice grain^2^.

The biodiversity and richness of the microbial community are considered to be critical to the integrity, function, and long-term sustainability of soil ecosystems, but they are usually diminished by agricultural perturbation^28^. In this study, long-term application of chemical NPK fertilizers resulted in a significantly lower rhizospheric bacterial richness index (chao1) (Table 2), the similar results were observed in bulk and rhizospheric soil by long-term fertilization experiments based on 454 pyrosequencing^2^ or Illumina Hiseq sequencing^17^ technologies, which mainly resulted from the lower pH subjected to long-term chemical fertilizers input^17^. Additionally, long-term application of composted manure either alone or together with chemical NPK fertilizers facilitated a higher soil bacterial biodiversity, which was significantly distinct from that shaped by long-term application of chemical NPK fertilizers or no fertilization (Table 2). These results supported previous reports from other long-term fertilizer trials^26,27^, probably due to the effects of manure application on soil physicochemical and biological properties, especially for soil pH^17^ and microbial biomass carbon^26^. All these results indicated that multiple bacterial groups dwell in the soil with long-term manure input, which could help for developing more balanced microbiome that allows rapid recovery from environmental stresses or natural perturbations, and then suppressing pathogens from flourishing^29^.

Any environmental change could alter the soil microbial community structure to some extent^30^. The PCoA based on the weighted UniFrac algorithm clearly demonstrated the variations among different fertilization regimes (Fig. 1). These four treatments were definitely separated either along the first component (PCoA1) or the second component (PCoA2) with respect to the bacterial communities. Above results were consistent with the observations by many long-term fertilization studies based on 454 pyrosequencing or Illumina Miseq sequencing methods^29,31^.

Analysis for phylum abundance indicated that *Proteobacteria, Acidobacteria, Chloroflexi* and *Nitrospirae* were the top 4 dominant phyla across all treatments or samples (Figs 2, S3), which was roughly in accordance with previous studies^2,32^. Analysis of variance and LEfSe jointly identified that the phyla *Actinobacteria* and *Planctomycetes* were most abundant in NPKM treatment (Figs 3, 4). *Actinobacteria* play key roles in organic matter decomposition and the humus formation process^33,34^, what’s more, which can produce variety of antibiotics^35^ to protect roots from pathogenic microorganisms^17^. Similar abundant tendency of *Planctomycetes* among treatments was reported by Chen *et al*.^24^ based on long-term fertilization trial, which accounting for seven genomes with distinct functional and transcriptional activities^36^. Meanwhile, the most abundant phylum *Verrucomicrobia* and family *Nitrospiraceae* observed in NPK treatment (Figs 3, 4). Similarly, Zhou *et al*.^37^ indicated that *Verrucomicrobia* was more abundant in long-term higher chemical fertilizer input than that in lower or no chemical fertilization, which involved in organic carbon utilization for its abundant transcripts^36^, while *Nitrospiraceae* was a potential keystone species for iron uptaking and nitrite oxidation^38^. Moreover, the most abundant *Acidobacteria* was detected in NF treatment (Figs 3, 4), supported previous studies^9,19,37^, which belong to the oligotrophic (or K-selected) group^39^, its function keeps obscure for the majority of them are ungrouped^40^. Additionally, the most abundant phylum *Proteobacteria*, class *Deltaproteobacteria* and order *Myxococcales* observed in M treatment (Figs 3, 4). *Deltaproteobacteria*, as one class of phylum *Proteobacteria*, the abundance of which tremendously increased probably leading to more abundant *Proteobacteria*. In fact, *Deltaproteobacteria* comprises a branch that predominantly composed of the genus *Haliangium*, including the relatives *Haliangium luteum*^41^ and *Haliangium ochraceum*^42^, which can produce *haliangicin*, a novel antifungal metabolite that can inhibit the growth of broad-spectrum fungi^43^. *Myxococcales*, as another abundant species in M treatment, had been reported to antagonize soil-borne plant pathogens^44^.

Previous studies had indicated that soil environmental factors affect the microbial community structure^28,45,46^. For example, Tu *et al*.^16^ confirmed that the available Ca and available Mg play important roles in shaping soil bacterial community composition in *Paulownia fortunei* plantations based on long-term fertilizer treatments. Wei *et al*.^18^ screened the SOC and TN as the main drivers that driven microbial community structure from six environmental variables (pH, SOC, TN, TP, IN and AK) based on 35 years of manure and chemical fertilizer application experiment. However, Wang *et al*.^17^ indicated that soil pH, organic matter and available P concentrations were the most important factors in shaping bacterial communities in the maize rhizosphere. In this study, CCA revealed that E-Mg^2+^, SOC, AK, AN, E-Ca^2+^ and SOC/TN were the key factors that driving the distribution and composition of the soil bacterial community (Fig. 5), while the Mantel test screened the SOC, AN, AP and E-Mg^2+^ as the key factors (Table 3). Different from previous studies, we concluded that E-Mg^2+^, SOC and AN were the key driving factors in shaping soil bacterial communities based on joint analysis of CCA and Mantel test. Guessing the above various results mainly caused by different soil parent materials, crop species, specific climates, etc. In spite of this, the carbon and nitrogen, as the two mainly important energy materials of soil microorganisms, is vital for shaping bacterial communities, which still were broadly supported by previous studies^15,18,47^.

**Table 3:** Spearman’s correlations (r) between the relative abundances of bacterial phyla and the environmental variables determined by the Mantel test.

Environmental variables	r	*P*
pH	0.3801	0.010
SOC	0.4972	0.001
AN	0.4026	0.008
AP	0.4724	0.002
AK	0.2805	0.037
E-Ca^2+^	0.3305	0.015
E-Mg^2+^	0.6686	0.001
SOC/TN	0.2270	0.059

SOC, soil organic carbon; AN, alkali-hydrolyzable nitrogen; AP, available phosphorus; AK, available potassium; E-Ca^2+^, exchangeable Ca^2+^; E-Mg^2+^, exchangeable Mg^2+^; SOC/TN, the ratio of soil organic carbon to soil total nitrogen.

## Conclusions

Long-term various fertilization regimes shaped different bacterial community structures in the rhizosphere of reddish paddy soil. Interestingly, balanced using of manure and chemical fertilizers not only increased the organic material pools and nutrient availability but also enhanced the biodiversity of the rhizospheric bacterial community. Moreover, balanced fertilization regime significantly increased the abundance of *Actinobacteria*, which involve in organic matter decomposition and the humus formation process, more importantly, it can produce various antibiotics to antagonize pathogenic microbes. Above advantages contribute to the sustainable development of agro-ecosystems. Additionally, CCA and the Mantel test clarified that E-Mg^2+^, SOC and AN are the key driving factors for shaping bacterial communities in the reddish paddy rhizospheric soil of Central South China.

## Materials and Methods

### Natural profile of experimental site

The long-term fertilization experiment was set up in 1982 at the Red Soil Experimental Station of the Chinese Academy of Agricultural Sciences, Qiyang County, Hunan Province, China (latitude, 26°45′N; longitude, 111°52′E; elevation, 120 m), which has a typical subtropical monsoon climate, with an average annual temperature of 18 °C, annual precipitation of 1250 mm, annual evaporation of 1470 mm, and 300 frost-free days. The paddy soil is derived from Quaternary red clay, the experiment mimicked the typical early rice-late rice double cropping system which is widely used in the hilly area of central south China, and the equivalent fertilization treatments were carried out in early and late rice, respectively. The initial properties of the plough layer (0∼20 cm) soil are listed as follows: pH, 5.97; soil organic matter, 1.98%; total nitrogen, 1.5 g kg^−1^; alkali-hydrolyzable nitrogen, 158.0 mg kg^−1^; total phosphorus, 0.5 g kg^−1^; available phosphorus, 9.6 mg kg^−1^; total potassium 14.2 g kg^−1^; and available potassium, 65.9 mg kg^−1^.

### Experimental design

The completely randomized block experiment included four treatments: no fertilizer application (NF); application of chemical NPK fertilizers (NPK); application of composted manure (M); and the combined application of composted manure and chemical NPK fertilizers (NPKM). Each treatment was replicated three times for a total of twelve plots, and each experimental plot was 27 m^2^ (1.8 m × 15 m). These plots were managed with separate irrigation inlets and drainage outlets. Chemical NPK fertilizers were applied as urea (46% N), calcium superphosphate (12% P_2_O_5_) and potassium chloride (60% K_2_O); manure was cow dung from the farm nearby and was composted completely before application, the muti-year average N, P_2_O_5_ and K_2_O content of which was 0.32%, 0.25% and 0.15%, respectively. All these fertilizers were used as basal fertilizers, and the application rates of the treatment plots are listed in Table 4.

**Table 4:** Fertilizer application rates of the treatment plots for each rice season (kg hm^−2^).

Treatment	Composted manure	Chemical fertilizers		
		N	P_2_O_5_	K_2_O
NF	0	0	0	0
NPK	0	72.5	56.3	33.8
M	22500	0	0	0
NPKM	22500	72.5	56.3	33.8

NF, no fertilization treatment; NPK, chemical NPK fertilizers treatment; M, composted manure treatment; NPKM, chemical NPK fertilizers plus composted manure treatment.

### Soil sampling and physicochemical analysis

To minimize the interference of recent fertilization, maximize the differences among different fertilization regimes and avoid interference from harvesting, sampling was performed prior to late rice harvesting in October 2016. A total of five plants were excavated randomly in each plot, and the rhizospheric soil was collected from each of the 5 plants according to the description by Han *et al*.^48^ and then pooled to form a composite sample. All samples were sealed in sterile plastic bags, packed on ice and transported to the laboratory immediately, and they were then sieved through 2-mm meshes and thoroughly homogenized after removing plant residues and gravel. Each sample was then divided into two parts: one was air-dried for analysis of soil properties, and the other was stored at −80 °C for DNA extraction.

The selected soil properties are listed in Table 2 and were measured following the description by Bao^49^. Briefly, soil pH was determined using a pH meter (FE20-FiveEasy^TM^ pH, Mettler Toledo, Germany) with a 1:5 ratio of soil to distilled water. Soil organic carbon (SOC) was determined using the vitriol acid-potassium dichromate oxidation method. Alkali-hydrolyzable N (AN) was measured by the alkali-hydrolysis and diffusion method. Available P (AP) was extracted with sodium bicarbonate and determined using the molybdenum-blue spectrophotometric method. Available K (AK) was extracted with ammonium acetate, and concentrations were determined using a flame photometer. Exchangeable Ca^2+^ (E-Ca^2+^) and Mg^2+^ (E-Mg^2+^) were extracted with ammonium acetate, and concentrations were determined using an atomic absorption spectrophotometer.

### DNA extraction, PCR and sequencing

For each sample, total genomic DNA was extracted from 0.5 g of fresh soil using the PowerSoil DNA Isolation Kit (MoBio Laboratories Inc., USA) according to the manufacturer’s instructions, and three successive DNA extractions of each sample were pooled before PCR to minimize DNA extraction bias. The DNA concentration and purity were monitored on 1% agarose gels, which were then stored at −40 °C for subsequent analysis.

An aliquot (50 ng) of DNA from each sample was used as a template for amplification^50^, and the V4-V5 hypervariable regions of bacterial 16S rRNAs (*Escherichia coli* positions 515–907)^51^ were amplified using the selected primers 515 F (5′-GTGCCAGCMGCCGCGGTAA-3′) and 907 R (5′-CCGTCAATTCCTTTGAGTTT-3′), which could minimize the overestimation of bacterial diversity due to intragenomic heterogeneity within 16S rRNA genes^52^. The forward and reverse primers were tagged with adapter, pad and linker sequences. Each barcode sequence was added to the reverse primer for the pooling of multiple samples in one run of HiSeq sequencing. All primers were synthesized by Sangon Biotech. co., Ltd. (Shanghai, China).

PCR amplification of each sample was performed in triplicate using a Gene Amp PCR-System 9700 (Applied Biosystems, Foster City, CA, USA) in a total volume of 30 μl, which contained 15 μl of Master Mix (2×), 0.5 μM the final concentrations of the forward and reverse primers, 10 ng of template DNA and double-distilled water up to 30 μl. The PCR was executed under the following conditions: initial denaturation at 94 °C for 5 min, followed by 30 cycles at 94 °C for 30 s, annealing at 55 °C for 30 s, extension at 72 °C for 30 s, with a final extension at 72 °C for 10 min. The triplicate PCR products were mixed equally after being purified with a Qiagen Gel Extraction kit (Qiagen, Germany), and then they were sent to Novogene Bioinformatics Technology Co., Ltd. (Beijing, China) for further sequencing based on the Illumina HiSeq2500 platform. The complete sequencing data were submitted to the NCBI Short Read Archive under Accession No. SRP135963.

### Processing of sequencing data

The raw sequences, after the barcodes and primers were trimmed, were assembled for each sample according to the unique barcode using QIIME^53^. The split sequences of each sample were merged using FLASH^54^, and low-quality sequences were discarded using QIIME. The retained sequences were analysed according to the UPARSE pipeline, using USEARCH and Perl scripts to generate an OTU table and to pick representative sequences^55^. Briefly, sequences with a quality score lower than 0.5, a length shorter than 200 nt or singletons were discarded, and the retained sequences were then assigned to operational taxonomic units (OTUs) based on 97% similarity after filtering chimeras. The representative sequences of each OTU were aligned and classified using the SILVA reference database for bacterial sequences^56^.

### Bioinformatic and statistical analysis

Rarefaction was performed to compare the relative levels of OTU richness across all soil samples at an OTU cut-off of 0.03. To correct the sampling effects, a randomly selected subset of 15,494 sequences for each sample were chosen for further bacterial community analysis. The OTU table was transformed into a suitable input file for further alpha and beta analyses using MOTHUR^57^. Species richness and biodiversity were estimated by the Chao1 estimator (Chao1), Shannon diversity index (Shannon) and Good’s nonparametric coverage (Coverage). Principal coordinate analysis (PCoA) and cluster analysis (unweighted pair group method using arithmetic averages, UPGMA) based on the calculated weighted UniFrac distance were used to explore the differences in the bacterial community structure among soil samples. Based on the Kruskal-Wallis (KW) sum-rank testing, linear discriminant analysis effect size (LEfSe) was performed to identify significantly different species (biomarkers) of bacterial taxa among groups, and then linear discriminant analysis (LDA) was performed to estimate the effect size of each biomarker^58^. Canonical correspondence analysis (CCA) and the Mantel test were performed to analyse the relationships between soil properties and dominant bacterial phyla, followed by 999 and 9,999 permutations to test the significance, respectively. An analysis of variance was performed using IBM SPSS 20.0 (SPSS Inc., USA).

## Electronic supplementary material

Supplementary materials

## References

[1] Shen JP, Zhang LM, Guo JF, Ray JL, He JZ (2010). Impact of long-term fertilization practices on the abundance and composition of soil bacterial communities in Northeast China. Appl. Soil Ecol..

[2] Chen D, Yuan L, Liu Y, Ji J, Hou H (2017). Long-term application of manures plus chemical fertilizers sustained high rice yield and improved soil chemical and bacterial properties. Eur. J. Agron..

[3] Savci S (2012). An agricultural pollutant: chemical fertilizer. Int. J. Environ. Sci. Dev..

[4] Singh H, Verma A, Ansari MW, Shukla A (2014). Physiological response of rice (*Oryza sativa* L.) genotypes to elevated nitrogen applied under field conditions. Plant Signal. Behav..

[5] Guo JH (2010). Significant acidification in major Chinese croplands. Science.

[6] Blanco-Canqui H, Schlegel AJ (2013). Implications of inorganic fertilization of irrigated corn on soil properties: lessons learned after 50 years. J. Environ. Qual..

[7] Hati KM, Mandal KG, Misra AK, Ghosh PK, Bandyopadhyay KK (2006). Effect of inorganic fertilizer and farmyard manure on soil physical properties, root distribution, and water-use efficiency of soybean in Vertisols of central India. Bioresour. Technol..

[8] Bhattacharyya R (2007). Long-term farmyard manure application effects on properties of a silty clay loam soil under irrigated wheat–soybean rotation. Soil Tillage Res..

[9] Sun R, Zhang X, Guo X, Wang D, Chu H (2015). Bacterial diversity in soils subjected to long-term chemical fertilization can be more stably maintained with the addition of livestock manure than wheat straw. Soil Biol. Biochem..

[10] Bandyopadhyay KK, Misra AK, Ghosh PK, Hati KM (2010). Effect of integrated use of farmyard manure and chemical fertilizers on soil physical properties and productivity of soybean. Soil Tillage Res..

[11] Ye J (2011). Effect of combined application of organic manure and chemical fertilizer on N use efficiency in paddy fields and the environmental effects in hang jiahu area. J. Soil Water Conserv..

[12] Aguilera J, Motavalli PP, Gonzales MA, Valdivia C (2012). Initial and residual effects of organic and inorganic amendments on soil properties in a potato-based cropping system in the Bolivian Andean Highlands. Am. J. Exp. Agr..

[13] Zhang X, Zhang Q, Liang B, Li J (2017). Changes in the abundance and structure of bacterial communities in the greenhouse tomato cultivation system under long-term fertilization treatments. Appl. Soil Ecol..

[14] Fierer N, Jackson RB (2006). The diversity and biogeography of soil bacterial communities. Proc. Natl. Acad. Sci. USA.

[15] Li C, Yan K, Tang L, Jia Z, Li Y (2014). Change in deep soil microbial communities due to long term fertilization. Soil Biol. Biochem..

[16] Tu J, Qiao J, Zhu Z, Li P, Wu L (2017). Soil bacterial community responses to long-term fertilizer treatments in Paulownia plantations in subtropical China. Appl. Soil Ecol..

[17] Wang Q (2017). Long-term fertilization changes bacterial diversity and bacterial communities in the maize rhizosphere of Chinese Mollisols. Appl. Soil Ecol..

[18] Wei M (2017). 35 years of manure and chemical fertilizer application alters soil microbial community composition in a Fluvo-aquic soil in Northern China. Eur. J. Soil Biol..

[19] Ai C (2018). Distinct responses of soil bacterial and fungal communities to changes in fertilization regime and crop rotation. Geoderma.

[20] Daquiado AR (2016). Pyrosequencing analysis of bacterial community diversity in long-term fertilized paddy field soil. Appl. Soil Ecol..

[21] Raaijmakers JM, Paulitz TC, Steinberg C, Alabouvette C, Moënne-Loccoz Y (2009). The rhizosphere: a playground and battlefield for soilborne pathogens and beneficial microorganisms. Plant Soil.

[22] Mendes R (2011). Deciphering the rhizosphere microbiome for disease-suppressive bacteria. Science.

[23] Peiffer J (2013). Diversity and heritability of the maize rhizosphere microbiome under field conditions. Proc. Natl. Acad. Sci. USA.

[24] Chen X (2017). Response of soil *phoD* phosphatase gene to long-term combined applications of chemical fertilizers and organic materials. Appl. Soil Ecol..

[25] Cooper JM, Warman PR (1997). Effects of three fertility amendments on soil dehydrogenase activity, organic C and pH. Can. J. Soil Sci..

[26] Li. J (2015). Soil microbial community structure and function are significantly affected by long-term organic and mineral fertilization regimes in the North China Plain. Appl Soil Ecol.

[27] Zhong W (2010). The effects of mineral fertilizer and organic manure on soil microbial community and diversity. Plant Soil.

[28] Zhao J (2014). Pyrosequencing reveals contrasting soil bacterial diversity and community structure of two main winter wheat cropping systems in China. Microb. Ecol..

[29] Pang G (2017). Trichoderma-enriched organic fertilizer can mitigate microbiome degeneration of monocropped soil to maintain better plant growth. Plant Soil.

[30] Burns RG (2013). Soil enzymes in a changing environment: Current knowledge and future directions. Soil Biol. Biochem..

[31] Liu W (2015). Changes in the abundance and structure of bacterial communities under long-term fertilization treatments in a peanut monocropping system. Plant Soil.

[32] Xu N, Tan G, Wang H, Gai X (2016). Effect of biochar additions to soil on nitrogen leaching, microbial biomass and bacterial community structure. Eur. J. Soil Biol..

[33] Kopecky J (2011). Actinobacterial community dominated by a distinct clade in acidic soil of a waterlogged deciduous forest. FEMS Microbiol. Ecol..

[34] Sun J, Zhang Q, Zhou J, Wei Q (2014). Pyrosequencing technology reveals the impact of different manure doses on the bacterial community in apple rhizosphere soil. Appl. Soil Ecol..

[35] Bull AT, Stach JE, Ward AC, Goodfellow M (2005). Marine actinobacteria: perspectives, challenges, future directions. Antonie Van Leeuwenhoek.

[36] Li M, Jain S, Dick GJ (2016). Genomic and transcriptomic resolution of organic matter utilization among deep-Sea bacteria in Guaymas basin hydrothermal plumes. Front. Microbiol..

[37] Zhou J (2015). Influence of 34-years of fertilization on bacterial communities in an intensively cultivated black soil in northeast China. Soil Biol. Biochem..

[38] Baker BJ (2013). Community transcriptomic assembly reveals microbes that contribute to deep-sea carbon and nitrogen cycling. ISME J..

[39] Fierer N, Bradford MA, Jackson RB (2007). Toward an ecological classification of soil bacteria. Ecology.

[40] Barns SM, Cain EC, Sommerville L, Kuske CR (2007). Acidobacteria phylum sequences in uranium-contaminated subsurface sediments greatly expand the known diversity within the phylum. Appl. Environ. Microbiol..

[41] Fudou R, Iizuka T, Yamanaka S (2001). Haliangicin, a novel antifungal metabolite produced by a marine myxobacterium: 1. Fermentation and biological characteristics. J. Antibiot..

[42] Kundim BA (2003). New haliangicin isomers, potent antifungal metabolites produced by a marine myxobacterium. J. Antibiot..

[43] Qiu M (2012). Application of bio-organic fertilizer can control Fusarium wilt of cucumber plants by regulating microbial community of rhizosphere soil. Biol. Fert. Soils.

[44] Taylor WJ, Draughon FA (2001). *Nannocystis exedens*: A potential biocompetitive agent against *aspergillus flavus* and *Aspergillus parasiticus*. J. Food Protect..

[45] Shen Z (2014). Deep 16S rRNA pyrosequencing reveals a bacterial community associated with banana *Fusarium* wilt disease suppression induced by bioorganic fertilizer application. PLoS One.

[46] Xun W (2015). Environmental conditions rather than microbial inoculum composition determine the bacterial composition, microbial biomass and enzymatic activity of reconstructed soil microbial communities. Soil Biol. Biochem..

[47] Xiao E (2016). Correlating microbial community profiles with geochemical conditions in a watershed heavily contaminated by an antimony tailing pond. Environ. Pollut..

[48] Han C, Zhong W, Shen W, Cai Z, Liu B (2013). Transgenic Bt rice has adverse impacts on CH_4_ flux and rhizospheric methanogenic archaeal and methanotrophic bacterial communities. Plant Soil.

[49] Bao, S. D. Analytical Methods of Soil Agrochemistry, 3rd edition. ChinaAgricultural Press, Beijing (In Chinese) (1999).

[50] Shen C (2013). Soil pH drives the spatial distribution of bacterial communities along elevation on Changbai mountain. Soil Biol. Biochem..

[51] Biddle JF, Fitz-Gibbon S, Schuster SC, Brenchley JE, House CH (2008). Metagenomic signatures of the Peru Margin subseafloor biosphere show a genetically distinct environment. Proc Natl Acad Sci USA.

[52] Sun DL, Jiang X, Wu QL, Zhou NY (2013). Intragenomic heterogeneity of 16S rRNA genes causes overestimation of prokaryotic diversity. Appl. Environ. Microb..

[53] Caporaso JG (2010). QIIME allows analysis of high-throughput community sequencing data. Nat. Methods.

[54] Magoc T, Salzberg SL (2011). FLASH: fast length adjustment of short reads to improve genome assemblies. Bioinformatics.

[55] Edgar RC (2013). UPARSE: highly accurate OTU sequences from microbial amplicon reads. Nat. Methods.

[56] Pruesse E (2007). SILVA: a comprehensive online resource for quality checked and aligned ribosomal RNA sequence data compatible with ARB. Nucleic Acids Res..

[57] Schloss PD (2009). Introducing mothur: open-source, platform-independent, community-supported software for describing and comparing microbial communities. Appl. Environ. Microb..

[58] Segata N (2011). Metagenomic biomarker discovery and explanation. Genome Biol..

