# Supplementary material for: Long-term combined application of manure and chemical fertilizer sustained higher nutrient status and rhizospheric bacterial diversity in reddish paddy soil of Central South China
Source: Sci Rep. 2018 Nov 8;8:16554. doi: 10.1038/s41598-018-34685-0 (PMC6224536; doi:10.1038/s41598-018-34685-0)
Supplement: Supplementary file 1 — Supplementary materials [file 41598_2018_34685_MOESM1_ESM.docx]

Long-term combined application of manure and chemical fertilizer sustained higher nutrient status and rhizospheric bacterial diversity in reddish paddy soil of Central South China

Xinwei Cui^1,3^, Yangzhu Zhang^1^, Jusheng Gao^2^, Fuyuan Peng^3^＆Peng Gao^3^

^1^College of Resources and Environment, Hunan Agricultural University, Changsha 410128, China. ^2^Red Soil Experimental Station of Chinese Academy of Agricultural Sciences, Qiyang, Hunan 426182, China. ^3^Institute of Agro-Environment and Ecology, Hunan Academy of Agricultural Sciences, Changsha 410125, China.

***Corresponding author:** Yangzhu Zhang & Jusheng Gao**.**

**E-mail address:** zhangyangzhu2006@163.com & gjusheng@163.com

**Journal of submission:** Scientific Reports

**Article type:** Original research

**Supplementary materials**

| **Table S1. Sequencing data and OTUs across treatments.** | | | |
| --- | --- | --- | --- |
| Treatment | Raw paired reads | Quality filtered reads | No. of OTU |
| NF1 | 86,686 | 54,355 | 2453 |
| NF2 | 94,730 | 53,344 | 2462 |
| NF3 | 87,291 | 48,797 | 2388 |
| NPK1 | 98,248 | 57,123 | 2290 |
| NPK2 | 63,555 | 50,555 | 2279 |
| NPK3 | 64,609 | 41,991 | 2312 |
| M1 | 66,673 | 42,562 | 2446 |
| M2 | 88,100 | 55,311 | 2499 |
| M3 | 55,639 | 41,702 | 2454 |
| NPKM1 | 94,553 | 54,320 | 2616 |
| NPKM2 | 93,132 | 49,748 | 2446 |
| NPKM3 | 92,682 | 51,528 | 2485 |
| Raw paired reads, raw sequencing data; Quality filtered reads, clean data after filtering and removing chimerical sequences, singletons, replicates and chimeras; No. of OUT, generated by clustering quality filtered reads based on 97% similarity. NF, no fertilization treatment; NPK, chemical NPK fertilizers treatment; M, composted manure treatment; NPKM, chemical NPK fertilizers plus composted manure treatment; 1, 2, 3 indicated replication. | | | |

**
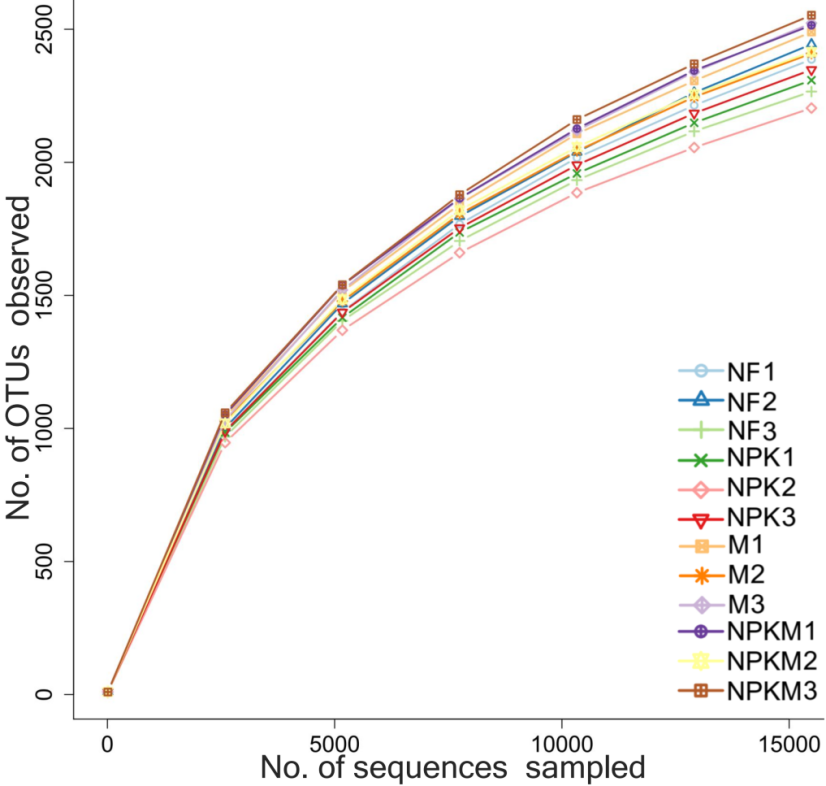
**

**Figure S1. Rarefaction curves of bacteria depicting the effect of 3% dissimilarity on the number of OTUs identified in the 12 soil samples.** Note: NF, no fertilization treatment; NPK, chemical NPK fertilizers treatment; M, composted manure treatment; NPKM, chemical NPK fertilizers plus composted manure treatment; 1, 2, 3 indicated replication.


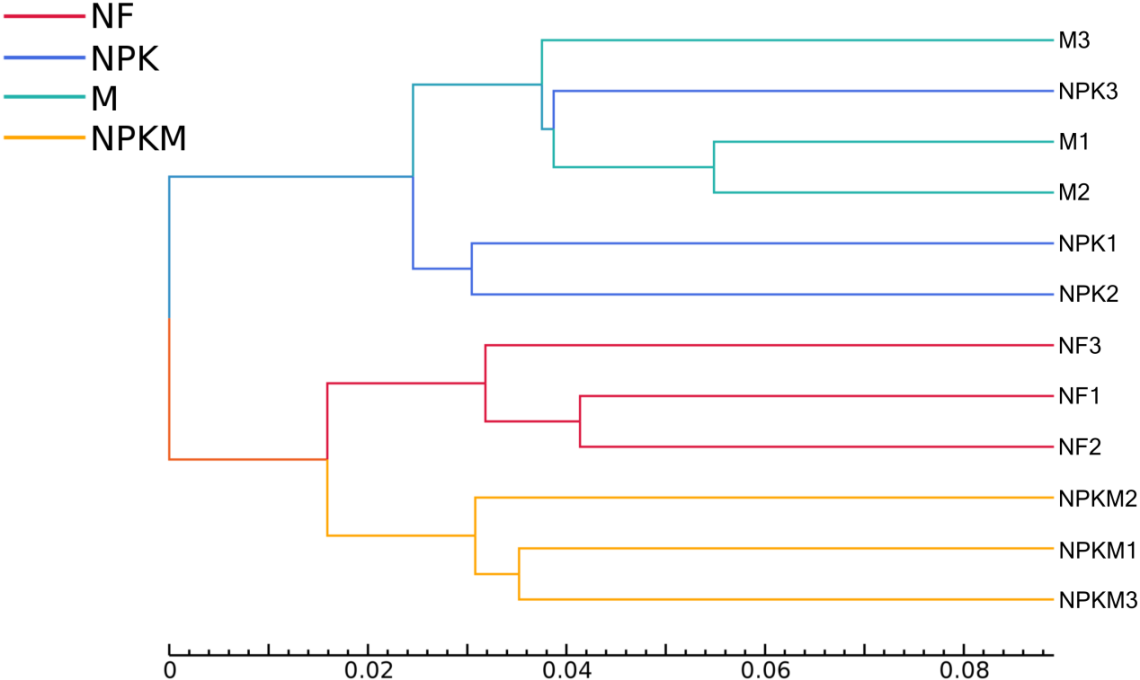


**Figure S2. Clustering of phylogenetic relationships of bacterial communities based on the weighted UniFrac similarity index of OTUs abundance.** Note: NF, no fertilization treatment; NPK, chemical NPK fertilizers treatment; M, composted manure treatment; NPKM, chemical NPK fertilizers plus composted manure treatment; 1, 2, 3 indicated replication.


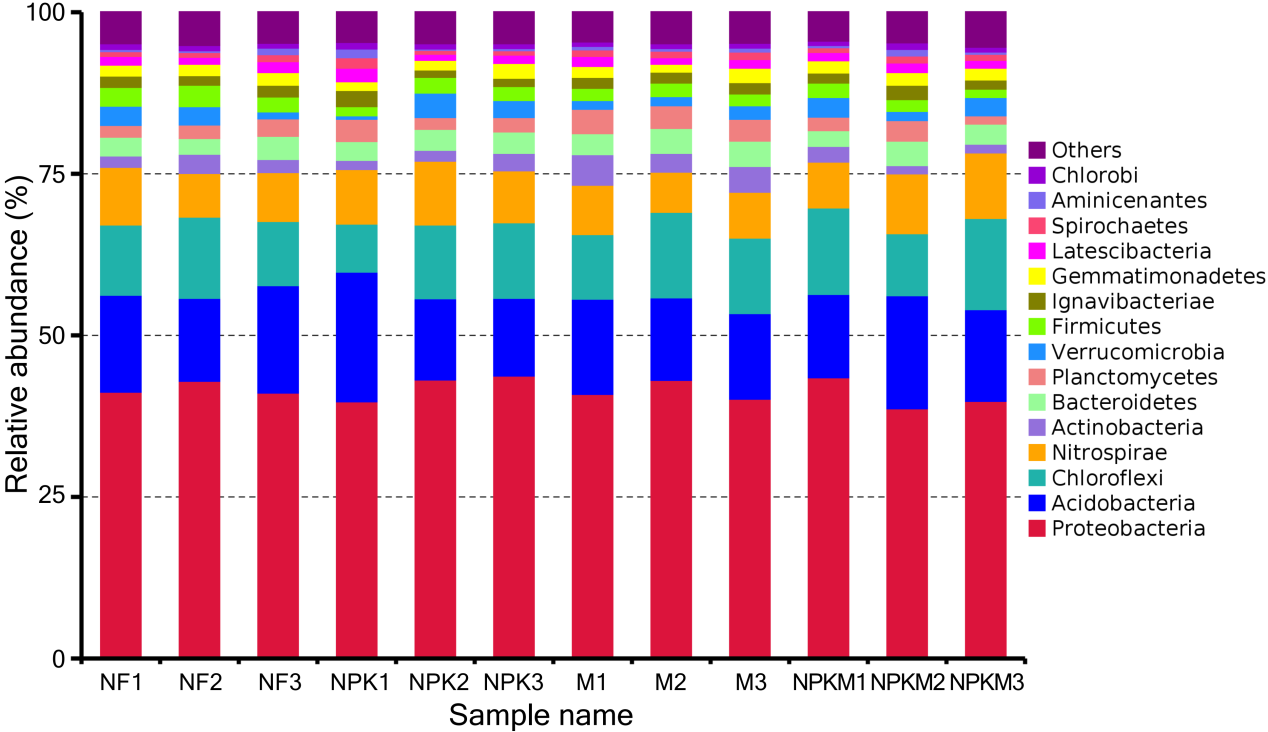


**Figure S3. The relative abundances (%) of the top 15 bacterial phyla present in all of the soil samples.** Note: NF, no fertilization treatment; NPK, chemical NPK fertilizers treatment; M, composted manure treatment; NPKM, chemical NPK fertilizers plus composted manure treatment; 1, 2, 3 indicated replication.
